# Apolipoprotein E, cognitive function, and cognitive decline among older Taiwanese adults

**DOI:** 10.1371/journal.pone.0206118

**Published:** 2018-10-19

**Authors:** Megan Todd, Lisa Schneper, Sarinnapha M. Vasunilashorn, Daniel Notterman, Michael T. Ullman, Noreen Goldman

**Affiliations:** 1 Columbia Aging Center, Columbia University, New York, New York, United States of America; 2 Department of Molecular Biology, Princeton University, Princeton, New Jersey, United States of America; 3 Division of General Medicine and Primary Care, Beth Israel Deaconess Medical Center and Harvard Medical School, Boston, Massachusetts, United States of America; 4 Department of Neuroscience, Georgetown University, Washington, D.C., United States of America; 5 Office of Population Research and Woodrow Wilson School of Public and International Affairs, Princeton University, Princeton, New Jersey, United States of America; Duke University, UNITED STATES

## Abstract

Apolipoprotein E (APOE) genotype is believed to play a role in the onset of dementia, though less is known about its relationship with non-pathogenic age-related cognitive decline. We assessed whether APOE was a risk factor for cognitive decline among older Taiwanese adults using nationally representative data. General cognition was measured longitudinally over eleven years; domain-specific cognitive assessments of working memory, declarative learning and three aspects of attention (executive function, alerting, and orientation) were performed once. Having at least one risky APOE allele was associated with more rapid longitudinal cognitive decline compared to those with no risky alleles. Some evidence from the cross-sectional analysis of domain-specific cognitive assessments suggested that APOE genotype may be more closely associated with working memory and declarative learning than with attention. Most genetic studies of cognition include only populations of European descent; extension is crucial. This study confirmed the association between APOE genotype and the rate of cognitive decline in a predominantly Han Chinese population. Additional studies on diverse populations are warranted.

## Introduction

Dementia and cognitive decline are major public health concerns that will only become bigger and more costly with the aging of the world’s population. The estimated current prevalence of dementia among those aged 60 and above is approximately 5–7% in regions around the world, and the number of people suffering from dementia is expected to double every twenty years if current trends continue [1]. Even without a diagnosable disease, cognitive decline is a hallmark of aging. In fact, a substantial portion of late life cognitive decline cannot be accounted for by common neurodegenerative pathologies, such as Alzheimer’s disease and cerebrovascular disease [2]. Age-related cognitive decline has a major impact on quality of life, and carries high societal and personal costs [3]. There is extensive heterogeneity in cognitive decline among older adults [4], and the determinants of these varying trajectories are not well understood.

Genetic factors are believed to be major risk factors for the development of Alzheimer’s disease. Best known is the ε4 allele of the apolipoprotein E gene (APOE), a well-established genetic risk factor for late-onset (after age 65) Alzheimer’s disease [5,6]. The ε4 allele is estimated to explain 4–6% of the variance in Alzheimer’s disease [7], and is expressed in more than half of Alzheimer’s disease patients [8]. APOE regulates cholesterol metabolism and is believed to modulate the clearance of amyloid-beta, the accumulation of which is a hallmark of Alzheimer’s disease [9,10]. The precise biological mechanism linking APOE genotype and Alzheimer’s disease, however, is not well-understood [8,11].

There is some evidence that APOE is associated with age-related cognitive decline that is not attributable to Alzheimer’s disease. Several studies have found an association between ε4 status and cognitive ability among those not suffering from dementia. Among non-demented Dutch [12] and white American [13] study participants, APOE genotype predicted memory scores. Wisdom et al.’s 2011 meta-analysis of non-demented individuals [14] found that APOE ε4 carriers performed worse than non-carriers on measures of episodic memory, executive functioning, and overall cognitive ability. Several studies have found evidence that APOE genotype is associated with change in cognitive ability as well. Studies of predominantly white Americans (which excluded those with Alzheimer’s disease or mild cognitive impairment) found more rapid longitudinal declines in memory among ε4 carriers compared to non-carriers [13,15]. A genome-wide association study (GWAS) of non-demented British and Swedish participants implicated APOE in longitudinal declines across a number of cognitive tests of fluid-type intelligence [3]. However, several authors [11,16,17] argue that any association between APOE and cognitive decline is largely or entirely attributable to pre-clinical Alzheimer’s disease, and thus APOE may not play a role in non-pathogenic decline.

Most genetic studies of cognition have been conducted on individuals of European ancestry, despite evidence that risk genes may act differently in different populations. For example, one US-based study found that APOE was associated with Alzheimer’s disease only among white, but not black or Hispanic, respondents [18]; another US study found a similar effect of APOE for predicting Alzheimer’s disease in white and black respondents, but substantial race differences in the effect size of other risk genes [19]. If the association between APOE genotype and Alzheimer’s disease risk varies by race/ethnicity, then this may be the case for age-related cognitive decline as well, though no study of which we are aware has directly addressed this question.

There is a critical need to examine the relationship between APOE genotype and cognition in non-Western populations. Replication of an association in multiple populations of different ethnicities strengthens the case that a particular genetic variant is responsible for the trait of interest. Questions remain about whether genetic risk factors found in European populations also hold in Asian populations, and research has thus far been limited. Several studies of Alzheimer’s disease in Han Chinese populations have confirmed a link between Alzheimer’s disease and APOE genotype [20,21], however the prevalence of the APOE ε4 allele among those diagnosed with Alzheimer’s disease has been found to vary substantially across geographic regions [22], suggesting that APOE genotype may be a more important determinant of Alzheimer’s disease risk among certain populations.

As for white European-ancestry populations, results on APOE and non-pathogenic cognitive decline in Asian populations have been mixed. In a Korean population, APOE genotype was not associated with age-related cognitive decline [23] (though it was associated with Alzheimer’s disease risk [24]). By contrast, studies of Chinese elders found that APOE was associated with age-related cognitive decline [25], as well as cross-sectional cognitive ability [26].

In this study, we examined whether APOE genotype was associated with age-related cognitive decline among cognitively healthy older Taiwanese adults. A major strength of this study is the richness of the cognitive assessments. A summary ten-item cognitive measure meant to reflect general cognition was assessed longitudinally, up to five times per respondent over the course of eleven years. In addition, in the final wave of data collection, respondents completed a number of detailed cognitive tasks designed to measure three aspects of attention (executive functioning, orienting, and alerting), working memory, and declarative learning, allowing for cross-sectional analysis of these individual cognitive domains.

## Materials and methods

The analyses described below were reviewed by the Institutional Review Board of Columbia University Medical Center and determined to be exempt (protocol # AAAQ4212).

### Data

The Taiwan Longitudinal Study of Aging (TLSA) is an ongoing nationally representative study of Taiwanese adults aged 50 or older. Begun in 1989, TLSA has conducted follow-up surveys every 3–4 years, with refresher samples of younger individuals added in each wave. TLSA asked respondents detailed questions about their health, including a ten-question general cognitive assessment.

A random subset of the TLSA sample was recruited for the Social Environment and Biomarkers of Aging Study (SEBAS), which was conducted in 2000, 2006, and 2011. SEBAS participants were asked to complete a hospital examination that allowed for collection of an extensive set of biomarkers, including deoxyribonucleic acid (DNA; from blood), in 2000 and 2006. Some of the selected TLSA participants (n = 111 in 2000 and n = 32 in 2006) were deemed ineligible for the SEBAS examination due to poor health. See Chang [27] for additional details of SEBAS sample construction. The cognitive assessment from TLSA was replicated in SEBAS waves; thus, comparable assessments were made in 2000, 2003, 2006, 2007, and 2011.

There were 1,420 unique respondents who completed the SEBAS examination in 2000 (n = 1,023) and/or 2006 (n = 1,036) and were asked to give DNA samples. Of these, 8 respondents did not have valid APOE data because the respondent didn’t consent to store DNA (n = 7), or the respondent refused blood collection (n = 1).

For the longitudinal analysis of the summary cognitive measure, we did not include respondents with missing demographic information on sex (n = 0), age (n = 0), or education (n = 23) or who didn’t complete at least one general cognitive assessment between 2000 and 2011 (n = 10). This resulted in an analytic sample size of 1,379. Most respondents (68%) participated in all available cognitive assessments, meaning 2000, 2003, 2006, 2007, and 2011 for respondents who joined SEBAS in 2000, and 2006, 2007, and 2011 for those who joined SEBAS in 2006.

Of those respondents in the longitudinal analysis, 985 participated in the 2011 SEBAS survey. However, not all had a valid performance measure for at least one of the detailed cognitive tasks measured in 2011. Some participants (n = 65) were interviewed by proxy (and thus did not complete the cognitive portion of the survey) due to serious illness (n = 32), deafness/being hard of hearing (n = 17), mental illness/senility (n = 11), inability to comprehend the survey (n = 2), being unwilling to complete the survey (n = 1) or other reasons (n = 2). An additional 111 respondents declined to participate in the cognitive assessment or failed to complete any valid trials for any of the tasks. Thus, there were 809 respondents with information from at least one of the detailed cognitive tasks. Because not every respondent had valid performance measures for all of the tasks, the sample size varied between 724 and 793 for these analyses.

Due to the nature of the sample construction, those included in our analyses were likely healthier than the general SEBAS population. We explore health and mortality selection in S1 Appendix.

### Measures

#### Summary cognitive measure

Overall cognition was assessed in 2000, 2003, 2006, 2007 and 2011 with ten cognitive and memory tasks, listed in Table 1. The tasks were derived from the Short Portable Mental Status Questionnaire [28], the Rey Auditory Verbal Learning Test [29,30], and a modified Digits Backward test from the Wechsler Adult Intelligence Scale [31]. Many of the tasks correspond to items used in the Chinese versions of the Mini-Mental Status Examination [32,33]. See Chang et al. [27] for further details of the cognitive tasks. Following the practices of Herzog and Wallace [34], the ten tasks are summed to create an overall score ranging from 0 to 24. If a respondent did not answer a task, it was coded as incorrect. These tasks have been analyzed in this fashion in prior studies of this population [35,36]; however, this cognitive measure is not directly comparable to a validated cognitive scale used in other populations.

**Table 1:** Questions included in the longitudinal summary cognitive assessment.

Item	Max score	Source
Tell me your address	1	SPMSQ
What is today's date? (Year, month and day)	3	SPMSQ
What day of the week is it?	1	SPMSQ
How old are you this year?	1	SPMSQ
What is your mother's maiden name?	1	SPMSQ
Who is the current president?	1	SPMSQ
Who was the president before him?	1	SPMSQ
Serial 3s subtraction task (4 times, starting at 20)	4	SPMSQ
10-item recall task (dog, cloth, watermelon, etc.)	10	RAVL
5 numbers repeated in reverse order task	1	WAIS
**Total**	**24**	

SPMSQ = Short Portable Mental Status Questionnaire

RAVL = Rey Auditory Verbal Learning Test

WAIS = Wechsler Adult Intelligence Scale

#### Detailed cognitive measures

Detailed cognitive measures were collected from SEBAS respondents only in the 2011 wave, with a series of tasks designed to measure attention, working memory, and declarative learning. All detailed cognitive tasks were designed by the Brain and Language Lab at Georgetown University.

Three aspects of attention (executive function, alerting, and orientation) were assessed with an Attention Network Task (ANT) based on prior work by Fan et al. [37] and Costa et al. [38]. In this task, participants were shown a row of five arrows, and asked to indicate the direction of the central arrow. The four flanking arrows could be pointing the same way (congruent) as the middle arrow, or the opposite way (incongruent), and could be preceded by various cues indicating where the central arrow might appear. The task was repeated many times with variation in the congruency and cue. Executive function was measured as the conflict effect, the difference in response time between congruent and incongruent trials. The alerting effect was measured as the difference in performance between trials with a cue and no cue. The orienting effect was measured as the difference in performance between trials with a cue placed exactly where the middle arrow would appear and trials with a cue placed in the center of the screen.

Working memory was evaluated via an adapted N-back task [39]. In the N-back task, respondents were shown a series of single digits, then asked whether the current digit was the same as the digit shown N items ago. For example, a respondent might be shown the following sequence of numbers: 2-5-3; the correct answer for the 1-back task would be 5, while the correct answer for the 2-back test would be 2. SEBAS participants completed 1-back and 2-back tasks. Further details of the N-back task are available in [40].

Declarative learning was assessed by a task designed by the Brain and Language Lab [41], wherein respondents were shown images of real and novel (imaginary) objects. After a delay of several minutes, the respondents were shown another set of images, some new and some repeated, and asked whether they had previously seen the object. Scores for declarative learning were assessed from the recognition phase of the task, that is, the period after the delay.

Performance was assessed by calculating response time for executive function, alerting, and orienting, and D’ scores for the N-back and declarative learning tasks. D’ is a measure of accuracy that quantifies discrimination between signal and noise. A D’ of zero indicates chance performance, higher positive values indicate better discrimination, and negative values indicate reverse discrimination (effective range -4.6 to 4.6). For details see Stanislaw and Todorov [42].

#### Genotyping

APOE genotype was determined by Union Clinical Laboratories in Taipei, Taiwan as described in Vasunilashorn et al. [43]. Briefly, DNA was extracted from whole blood, then amplified with polymerase chain reaction amplification refractory mutation system and polymerase chain reaction restriction fragment length polymorphism analysis.

### Analytic strategy

#### Longitudinal analysis

To model individuals’ change in cognitive ability over time, we used age-based growth curves. This allowed us to determine whether those with at least one ε4 allele followed steeper trajectories of decline per year of age compared to those with no ε4 alleles. All models controlled for sex (by including an indicator variable for female), years of education (centered at six years, close to the mean), and, implicitly, age.

For respondents genotyped in 2000, we used all cognitive test measures taken in 2000 or later, for up to five cognitive measures (2000, 2003, 2006, 2007, 2011). For those not genotyped until 2006, we used only cognitive test results available in 2006 or later.

#### Cross-sectional analysis

For the cross-sectional analysis of specific cognitive domains, we used linear regression models to examine the association between APOE genotype and the detailed cognitive assessments taken in 2011. As with the longitudinal analysis, all models controlled for sex, education, and age.

## Results

### Longitudinal analysis of summary cognitive measures

Descriptive statistics of the analytic sample are shown in Table 2. The sample, comprised of the original SEBAS study cohort as well as younger refresher samples added in later years, had an average age of 68 years in 2000, 67 years in 2006, and 70 in 2011. The respondents had relatively low educational attainment: just 6 years on average. Most respondents (72%) were genotyped based on a blood sample taken in 2000; the remaining respondents were genotyped from a 2006 blood sample. Respondents had up to five valid cognitive scores, measured in 2000, 2003, 2006, 2007, and 2011. Respondents had 3.6 cognitive scores on average; 80% of respondents had three or more. Further details of the cognitive scores and measures are shown in S1 Fig.

**Table 2:** Descriptive statistics of the longitudinal general cognition sample.

		Mean or %	SD	Median	N
Female		44.4%	—	—	1,379
Age					
	2000	68.0	8.4	68.0	976
	2003	70.4	8.1	70.0	858
	2006	66.8	10.1	65.0	1,102
	2007	67.3	10.0	66.0	1,100
	2011	69.8	9.3	68.0	919
Age when genotyped*		64.7	8.9	62.0	1,374
Year genotyped*					
	2000	71.7%	—	—	
	2006	28.3%	—	—	
Education, years		6.3	4.8	6.0	1,379
Summary cognitive score [range 0–24]					
	2000	16.6	3.6	17.0	976
	2003	15.4	3.9	16.0	858
	2006	16.4	3.7	17.0	1,102
	2007	16.2	3.9	17.0	1,100
	2011	15.9	4.0	17.0	919
Number of summary cognitive score measures		3.6	1.3	3.0	1,379
	One cognitive score	8.0%	—	—	
	Two cognitive scores	11.6%	—	—	
	Three cognitive scores	30.5%	—	—	
	Four cognitive scores	12.8%	—	—	
	Five cognitive scores	37.1%	—	—	
APOE genotype					1,379
	Two risk alleles	0.4%	—	—	
	One risk allele	13.9%	—	—	
	Zero risk alleles	85.8%	—	—	

* Date of genotyping was missing for 5 respondents.

Table 3 shows results from the age-based growth curve models of cognitive scores between 2000 and 2011. In these models, the intercept (cognitive score at age 65) and slope (change in cognitive score per year of age) were allowed to vary randomly above and beyond the systematic differences associated with covariates in the model. Model 1 shows the association between having at least one APOE ε4 allele and cognitive score, and Model 2 adds an ε4 status * age interaction. The risk associated with the ε4 allele was modeled as dominant, meaning one or more copies of the risky allele was assumed to convey risk. In this sample, very few respondents (0.4%) were homozygous for the ε4 allele, so the results from an additive risk model—wherein each additional ε4 allele conveys additional risk—were nearly identical to the dominant risk model (additive risk model results not shown). All models included sex and education as covariates.

**Table 3:** Coefficients and 95% confidence intervals from growth curve models of longitudinal cognitive score, 2000–2011.

	(1)	(2)
	Beta/95% CI	Beta/95% CI
Constant	16.666	16.674
	(16.487, 16.844)	(16.495, 16.853)
Age, centered at 65	-0.163	-0.154
	(-0.177, -0.149)	(-0.170, -0.139)
At least one ε4 allele	-0.055	-0.132
	(-0.393, 0.283)	(-0.474, 0.209)
At least one ε4 allele * age		-0.063
		(-0.103, -0.024)
SD(slope)	0.132	0.129
	(0.112, 0.156)	(0.109, 0.154)
SD(intercept)	1.651	1.657
	(1.522, 1.792)	(1.527, 1.798)
Corr(intercept, slope)	0.501	0.501
	(0.328, 0.641)	(0.326, 0.643)
SD(residual)	2.311	2.310
	(2.254, 2.370)	(2.253, 2.369)
Number of observations	4,955	4,955
Number of respondents	1,379	1,379
P-value from joint test of ε4 & ε4*age		0.006

Note: All models included sex (a female indicator variable) and years of education (centered at 6 years) as covariates.

Model 1 estimated cognitive score trajectories as a function of having at least one APOE risk allele (ε4). In this model, the mean cognitive score for a 65-year-old man with six years of education was 16.7 points (out of 24 total). Cognitive scores declined with age: each year of age was associated with a 0.16-point score reduction. The standard deviations of the intercept and slope shown in Model 1 indicate that there was statistically significant (p<0.05) respondent-level variation in the cognitive score at age 65 and in the annual rate of change in the cognitive score. The correlation between the intercept and slope, estimated at 0.50, indicates that respondents with lower baseline cognitive score were also more likely to have steeper declines in cognitive score with increasing age compared to respondents with higher baseline cognitive scores. Model 1 shows that APOE genotype was not significantly associated with cognitive score at age 65 (95% CI (-0.393, 0.283)).

Model 2 adds an APOE genotype*age interaction to test whether ε4 status was associated with a more rapid annual decline in cognition. The point estimates for the constant term and the age term were very similar to those in Model 1, and again APOE genotype was not significantly associated with cognitive score at age 65 (95% CI (-0.474, 0.209)). However, APOE genotype was significantly associated with the annual rate of decline in cognition. Respondents with at least one APOE ε4 allele had an additional .06-point decline in cognitive score per year of age compared to respondents with no ε4 alleles. Thus, the age-related decline in cognitive score was approximately 40% steeper for those carrying an ε4 allele (-0.22 points per year of age) than for those with no ε4 alleles (-0.16 points per year of age). To put this in context, a respondent with at least one ε4 allele experienced in five years the same decline in cognitive score that would be expected from about seven years of aging in a respondent with no ε4 alleles, on average.

### Cross-sectional analysis of detailed cognitive measures

Summary statistics of the sample included in the cross-sectional analyses are shown in Table 4. While the longitudinal analysis included respondents who were genotyped in 2000 or 2006 and contributed at least one summary cognitive measure, this cross-sectional analysis was further limited to respondents who completed at least one of the detailed cognitive assessments in 2011 (n = 809). The minimum age was higher in the cross-sectional analysis than the longitudinal analysis (58 vs. 53), though the average age of 2011 participants was about the same in the two analyses (69.3 vs 69.8 years), indicating that mortality and health selection played a role in participation in the detailed cognitive assessments (see S1 Appendix for more details).

**Table 4:** Descriptive statistics of the cross-sectional detailed cognition sample.

		Mean or %	SD	Med.	N
Female		46.8%	—	—	809
Year genotyped					
	2000	58.9%	—	—	
	2006	41.1%	—	—	
Age, 2011		69.3	9.1	67.0	809
Age when genotyped		61.2	7.0	59.0	806
Education, years		7.2	4.7	6.0	809
Summary cognitive score [range 0–24]					
	2011	16.4	3.4	17.0	800
Detailed cognitive measures, 2011		4.1	1.0	5.0	809
	1-back, D'	2.1	1.3	2.4	787
	2-back, D'	1.3	1.1	1.3	787
	Declarative learning, overall, D'	0.7	0.5	0.7	793
	Declarative learning, real objects only, D'	0.9	0.8	0.9	793
	Declarative learning, novel objects only, D'	0.4	0.4	0.4	793
	ANT: conflict effect, response time (ms)	48.6	75.5	47.8	724
	ANT: alerting effect, response time (ms)	-1.9	61.5	0.3	725
	ANT: orienting effect, response time (ms)	15.3	70.9	13.8	725
APOE genotype					809
	Two risk alleles	0.4%	—	—	
	One risk allele	13.5%	—	—	
	Zero risk alleles	86.2%	—	—	

The cross-sectional analyses of the detailed cognitive tasks assessed in 2011 are shown in Tables 5 and 6. Linear regression models were used to estimate whether domain-specific cognitive ability was associated with APOE genotype (Table 5), and whether APOE genotype modified the age-cognition relationship (Table 6). Columns 1–8 show the results for models of D’ for the N-back task (columns 1–2) and the declarative learning task (columns 3–5), and of response time differences for the attention tasks: conflict (executive function, column 6), alerting (column 7), and orienting (column 8). Each model also included a constant term, and sex and education covariates (coefficients not shown).

**Table 5:** Coefficients and 95% confidence intervals from cross-sectional linear regression models of detailed cognitive assessments, 2011: Regression models of detailed cognitive assessments on APOE genotype.

	(1) 1-back D'	(2) 2-back D'	(3) Decl. learn, all objects, D'	(4) Decl. learn, real, D'	(5) Decl. learn, novel, D'	(6) ANT: conflict, response time	(7) ANT: alerting, response time	(8) ANT: orienting, response time
	Beta (95% CI)	Beta (95% CI)	Beta (95% CI)	Beta (95% CI)	Beta (95% CI)	Beta (95% CI)	Beta (95% CI)	Beta (95% CI)
At least one APOE ε4 allele	-0.041	-0.248	-0.067	-0.131	-0.018	0.813	1.308	-10.563
	(-0.270, 0.187)	(-0.431, -0.064)	(-0.153, 0.019)	(-0.255, -0.006)	(-0.099, 0.063)	(-14.897, 16.522)	(-11.565, 14.181)	(-25.320, 4.194)
Age, centered at 65	-0.042	-0.044	-0.018	-0.028	-0.011	-0.491	-0.006	0.477
	(-0.051, -0.033)	(-0.052, -0.037)	(-0.022, -0.015)	(-0.033, -0.023)	(-0.014, -0.007)	(-1.162, 0.181)	(-0.553, 0.541)	(-0.151, 1.105)
N	787	787	793	793	793	724	725	725

**Table 6:** Coefficients and 95% confidence intervals from cross-sectional linear regression models of detailed cognitive assessments, 2011:: Regression models of detailed cognitive assessments on APOE genotype and APOE genotype*age.

	(1) 1-back D'	(2) 2-back D'	(3) Decl. learn, all objects, D'	(4) Decl. learn, real, D'	(5) Decl. learn, novel, D'	(6) ANT: conflict, response time	(7) ANT: alerting, response time	(8) ANT: orienting, response time
	Beta (95% CI)	Beta (95% CI)	Beta (95% CI)	Beta (95% CI)	Beta (95% CI)	Beta (95% CI)	Beta (95% CI)	Beta (95% CI)
At least one APOE ε4 allele	-0.010	-0.249	-0.057	-0.155	0.015	3.938	5.625	-5.831
	(-0.255, 0.236)	(-0.446, -0.053)	(-0.150, 0.036)	(-0.289, -0.020)	(-0.073, 0.102)	(-13.267, 21.144)	(-8.459, 19.710)	(-21.980, 10.317)
Age, centered at 65	-0.041	-0.044	-0.018	-0.029	-0.009	-0.391	0.131	0.627
	(-0.051, -0.031)	(-0.052, -0.036)	(-0.022, -0.014)	(-0.034, -0.023)	(-0.013, -0.006)	(-1.099, 0.317)	(-0.446, 0.707)	(-0.035, 1.289)
At least one APOE ε4 allele * age	-0.010	0.000	-0.003	0.007	-0.009	-0.826	-1.141	-1.250
	(-0.036, 0.017)	(-0.021, 0.022)	(-0.013, 0.007)	(-0.008, 0.021)	(-0.018, 0.000)	(-2.679, 1.027)	(-2.657, 0.376)	(-2.989, 0.488)
N	787	787	793	793	793	724	725	725

All models included age, sex, and years of education.

The cross-sectional results provided mixed findings regarding the effect of the ε4 allele. Having at least one ε4 allele of APOE was significantly associated with worse performance on the 2-back task at age 65 (0.25 worse D’; Table 5, column 2), and the real objects portion of the recognition phase from the declarative learning task (0.13 worse D’; Table 5, column 4). APOE genotype was not significantly associated with performance on the 1-back task or the novel objects portion of the declarative learning task (Table 5, columns 1 and 5), nor was it associated with response time for any of the three attention tasks (Table 5, columns 6–8). Age was significantly associated with worse performance on both N-back tasks and both the real and novel objects portions of the declarative learning task (Table 5, columns 1–5), but was not associated with response time for any of the attention tasks (Table 5, columns 6–8). The APOE genotype * age interaction was not statistically significant in any models (Table 6), suggesting that ε4 status may not alter the association between age and performance on these cognitive tasks.

## Discussion

The goal of this study was to test whether APOE genotype, which has been strongly implicated in Alzheimer’s disease, was associated with age-related cognitive decline among cognitively healthy older Taiwanese adults. In growth curve models using a general cognitive measure collected longitudinally, having at least one ε4 allele was associated with steeper declines in cognitive score per year of age. However, APOE genotype was not associated with baseline cognitive score in these models. In cross-sectional analyses of specific cognitive domains, there was some, but not conclusive, evidence that a risky APOE genotype may be associated with worse performance in the domains of working memory and declarative learning.

The lack of association between APOE genotype and baseline general cognitive score in our growth curve models is surprising given several studies that have shown APOE to predict cognitive performance among non-demented study participants [12–14]. However, the studies that did show an association tended to use domain-specific measures of cognition, such as memory [12–14] and executive functioning [14], rather than the general measure we used. It is possible that our general cognitive measure was too noisy or broad to pick up domain-specific effects.

Results from our cross-sectional analysis support the view that the APOE-cognition link may be domain-specific. We found evidence suggesting that APOE may be more closely linked to working memory and declarative learning than to attention, consistent with past work showing that certain cognitive domains may be more affected by APOE genotype than others [14,44]. Prior studies have found deficits in working memory among healthy carriers of the ε4 allele [45,46], suggesting that the effect of APOE on age-related cognitive decline could act via working memory. Declarative learning has been less studied in relation to APOE, but one early study also implicated APOE in age-related declarative learning impairment [47]. Still, our cross-sectional results were mixed, and it is possible that the statistically significant results we found were due to chance.

The ε4 allele of APOE has been found to be associated with longitudinal cognitive decline in non-demented study participants [3,15,48,49]. However, nearly all genetic studies have been conducted on populations of European descent, and there is uncertainty about whether these associations are present in other ethnic groups; the few prior studies on Asian populations have been inconsistent [23,24]. We confirmed the relationship found in studies of European-ancestry individuals that a risky APOE genotype was associated with steeper age-related cognitive decline in this population of Taiwanese older adults primarily of Chinese Han ancestry.

This study has several limitations. First, as is always the case, a larger sample would have been desirable; Liu et al. [50] suggested that small sample sizes could be responsible for the mixed findings on genetics and cognition in Asian populations. Still, our sample is larger than many of the studies Liu critiques. Second, selection bias is almost certainly at play since those respondents who experienced the most dramatic cognitive declines were less likely to participate in cognitive testing in later waves, either due to mortality or poor health. Thus, the risk associated with APOE genotype might be underestimated if those respondents unable to participate in later waves were more likely to have at least one ε4 allele than those who did participate. We were able to perform a simple test of this by replicating the analysis using only the healthiest and least healthy participants in our sample. We found that our main conclusions remained the same in these two subsamples. See S1 Appendix for details of this analysis and further discussion of selection. Third, the relatively low educational attainment of the respondents (about six years on average) raises questions about the generalizability of these findings to more educated populations, including more recent cohorts of older Taiwanese. Fourth, our measure of global cognition, although composed of items used in well-validated cognitive instruments, is not directly comparable to assessments from other studies. Still, we do not feel that is a major limitation. We intend our results to describe the relationship between APOE genotype and cognitive function, as measured in one particular way; certainly, other ways of measuring cognition are valid.

Finally, several authors argue that the association between APOE genotype and cognitive decline may be due to confounding from pre-clinical Alzheimer’s disease [11,16,17]. Because of the nature of our sample creation, it is unlikely that any of our respondents had Alzheimer’s disease or dementia. However, it is still possible that some were in the early pre-clinical stages of pathogenic decline; if this were the case, we might have incorrectly classified pathogenic decline as age-related decline. Similar to other similar studies, we were not able to rule out the possibility that pre-clinical Alzheimer’s disease was responsible for the association between APOE and cognitive change. The absence of individuals with Alzheimer’s disease or other dementias is a further limitation of this study; given the surprising lack of association between APOE genotype and baseline general cognitive score, it would be interesting to verify whether APOE genotype predicted Alzheimer’s disease in this sample. If not, it would provide evidence that genetic risk for cognitive decline—whether neuropathological or age-related—may be specific to a particular population or ethnicity.

Nevertheless, our study had several important strengths. Consistent, repeated testing allowed us to compare cognitive trajectories over 11 years in nearly 1400 respondents, and to examine a detailed assessment of individual cognitive domains on a subsample. Further, we conducted our study in a nationally representative sample of older Taiwanese adults, a population that has been largely neglected in the genetic study of cognition.

This study confirmed the association between APOE genotype and cognitive decline in a predominantly Han Chinese population of older adults. Additional research is warranted to better characterize the genetic determinants of age-related cognitive decline in diverse populations.

## Supporting information

S1 Fig Distribution of summary cognitive score measures in 2000, 2003, 2006, 2007, and 2011. (PDF)

S1 Appendix Mortality and health selection of the analytic sample. (PDF)
