# Supplementary material for: Apolipoprotein E, cognitive function, and cognitive decline among older Taiwanese adults
Source: PLoS One. 2018 Oct 19;13(10):e0206118. doi: 10.1371/journal.pone.0206118 (PMC6195295; doi:10.1371/journal.pone.0206118)

S1 Fig. Distribution of summary cognitive score measures in 2000, 2003, 2006, 2007, and 2011

The general cognitive score is a sum of ten items as shown in Table 1. Histograms of the summary cognitive score in each year are shown in S1 Figure, with a normal curve superimposed for reference. A sensitivity analysis that used robust standard errors reached the same conclusions as our original model (results not shown).

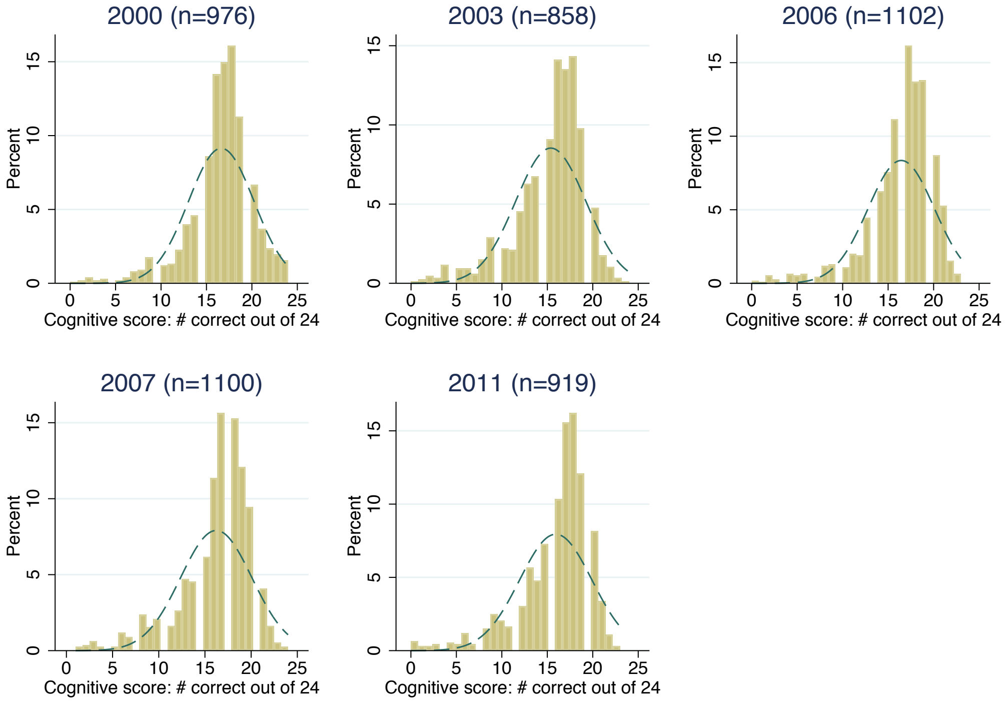

Supplement: S1 Fig — (PDF) [file pone.0206118.s001.pdf]
