# Supplementary material for: Apolipoprotein E, cognitive function, and cognitive decline among older Taiwanese adults
Source: PLoS One. 2018 Oct 19;13(10):e0206118. doi: 10.1371/journal.pone.0206118 (PMC6195295; doi:10.1371/journal.pone.0206118)
Supplement: S1 Appendix — (PDF) [file pone.0206118.s002.pdf]

S1 Appendix. Mortality and health selection of the analytic sample

The construction of the analytic sample was likely to result in respondents who were healthier than the underlying population. This selection occurred in a number of places:

1) Original participation in TLSA (from which the SEBAS sample is selected). Those who agree to participate in research studies are generally healthier, with lower mortality rates than the general population [e.g., 1].

2) Participation in the SEBAS examination. One component of SEBAS included a visit to a hospital for a physical exam with a blood draw. A number of respondents were excluded from this exam because of ill health (n=111 in 2000 and n=32 in 2006), i.e., living in an institution, being seriously ill, having a catheter or diaper, receiving kidney dialysis, or having a condition that precluded a blood draw.

3) Selection into the longitudinal sample. Some SEBAS participants (n=41) were excluded from the longitudinal sample due to refusing DNA storage, missing demographic information, or missing general cognitive assessments. It is possible that excluded respondents were in worse health than those included in the sample.

4) Selection into the cross-sectional sample. Because the detailed cognitive assessments were measured in 2011, all respondents in this sample must have survived until 2011. They also must have been healthy enough to participate in the 2011 survey, including the lengthy cognitive supplement with detailed domain-specific measures.

To assess the extent of mortality and health selection in our samples, we compare mortality rates, self-rated health, and the number of general cognitive assessments completed by all SEBAS participants, those included in the longitudinal sample, those included in the cross-sectional sample, and those SEBAS participants who

were excluded from both the longitudinal and cross-sectional samples. Note that mortality was not a reason for exclusion from the longitudinal analysis, so long as the participant had completed at least one general cognitive assessment. Results are shown in S1 Appendix Table 1.

S1 Appendix Table 1. A comparison of mortality, self-rated health, and number of cognitive assessments for all SEBAS participants and the analytic samples

	(1) All SEBAS participants (n=1420)	(2) Included in longitudinal sample (n=1379)	(3) Included in cross-sectional sample (n=809)	(4) Excluded from both samples (n=41)
Mortality				
Died by end of 2008	18.1%	17.4%	0.0%	41.5%
Died by end of 2011	25.6%	24.8%	0.1%	53.7%
Average self-rated health (scale: 1 = poor to 5 = excellent)				
2006	3.1	3.1	3.2	3.1
2011	3.1	3.1	3.2	2.8
Average number of general cognitive assessments				
	3.6	3.6	4.1	2.5

Mortality was determined from death registration data from the Ministry of Health and Welfare, and from survey workers who learned of a respondent's death when attempting to administer a survey. Compared to all SEBAS participants, those included in the longitudinal sample had slightly lower mortality rates as of 2008 and 2011 (S1 Appendix Table 1, columns 1-2). By definition, those included in the 2011 cross-sectional sample had zero deaths by 2008, with only one respondent dying between the administration of the 2011 survey and the end of the calendar year (column 3). By contrast, the 41 respondents excluded from both longitudinal and cross-sectional samples had substantially higher mortality rates than all SEBAS participants (column 4), as expected.

Self-rated health was determined by the following question: "regarding your current state of health, do you feel it is excellent (5), good (4), average (3), not so good (2), or poor (1)?" The longitudinal sample was comparable to all SEBAS participants on self-rated health (columns 1-2), while the cross-sectional sample had slightly higher self-rated health (column 2) and the excluded respondents had slightly lower self-ratings (column 4).

Likewise, the longitudinal sample has the same average number of general cognitive assessments as all SEBAS participants (columns 1-2, out of five total), while the cross-sectional sample had more assessments (column 3) on average and the excluded participants had fewer (column 4).

Based on this analysis, the longitudinal sample was slightly healthier than SEBAS participants as whole, but in general quite comparable. The cross-sectional sample had more substantial health and mortality selection forces at play.

To assess whether health selection might be affecting our results, we reran our primary longitudinal analysis on two subsamples, constructed to include the most and least healthy participants (S1 Appendix Table 2). The healthy sample consisted of respondents who participated in all five general cognitive assessments and had not died by the end of 2011. The unhealthy sample consisted of respondents who had died by the end of 2011. This sensitivity analysis did not change our primary conclusion: APOE ε4 status was not associated with baseline cognitive score, but was associated with a more rapid decline in cognitive score per year of age, in both the healthy and unhealthy samples. The point estimate of the ε4 genotype*age interaction term among the healthiest sample (column 3) is quite similar to the main effect estimated in Table 3. The point estimate for the unhealthiest group is approximately double the magnitude (column 4), but the 95% confidence intervals are quite wide, and include the main effect from Table 3.

S1 Appendix Table 2. Sensitivity analysis: Coefficients and 95% confidence intervals from growth curve models of longitudinal cognitive score on healthy and unhealthy subsamples, 2000-2011

	Healthiest sample		Unhealthiest sample	
	(1)	(2)	(3)	(4)
	Beta/95% CI	Beta/95% CI	Beta/95% CI	Beta/95% CI
Constant	17.263	17.235	17.105	16.896
	(16.997, 17.530)	(16.968, 17.502)	(16.509, 17.702)	(16.292, 17.500)
Age, centered at 65	-0.178	-0.167	-0.209	-0.187
	(-0.198, -0.158)	(-0.189, -0.145)	(-0.253, -0.166)	(-0.232, -0.142)
At least one ε4 allele	-0.327	-0.180	-0.809	0.507
	(-0.814, 0.160)	(-0.679, 0.319)	(-1.920, 0.302)	(-0.988, 2.002)
At least one ε4 allele * age		-0.073		-0.165
		(-0.129, -0.016)		(-0.293, -0.038)
SD(slope)	0.116	0.113	0.166	0.152
	(0.088, 0.152)	(0.085, 0.150)	(0.104, 0.263)	(0.090, 0.258)
SD(intercept)	1.547	1.547	2.506	2.476
	(1.385, 1.728)	(1.386, 1.727)	(1.989, 3.157)	(1.975, 3.104)
Corr(intercept, slope)	0.253	0.273	-0.313	-0.254
	(-0.049, 0.514)	(-0.039, 0.537)	(-0.643, 0.115)	(-0.624, 0.211)
SD(residual)	2.257	2.257	2.921	2.918
	(2.183, 2.334)	(2.183, 2.334)	(2.721, 3.135)	(2.719, 3.131)
Number of observations	2,555	2,555	799	799
Number of respondents	511	511	342	342
P-value from joint test of ε4 & ε4*age		0.017		0.015

References

1. Keyes KM, Rutherford C, Popham F, Martins SS, Gray L. How Healthy Are Survey Respondents Compared with the General Population? Epidemiol Camb Mass. 2018;29: 299–307. doi:10.1097/EDE.0000000000000775
